# Developing disruptive mobility scenarios for rural areas. Participatory mobility scenario building in a Belgian village for the year 2050

**DOI:** 10.1186/s12544-022-00555-0

**Published:** 2022-07-22

**Authors:** Sara Tori, Jesse Pappers, Imre Keserü

**Affiliations:** 1 MOBI Mobility, Logistics and Automotive Technology Research Centre, Vrije Universiteit Brussel, Pleinlaan 2, 1050 Brussels, Belgium; 2 BUTO Business Technologies and Operations, Vrije Universiteit Brussel, Pleinlaan 2, 1050 Brussels, Belgium

**Keywords:** Scenario building, Wild cards, Visions, Participation, Rural mobility

## Abstract

**Background:**

Historically, quantitative forecasting methods have been used in transport planning. As forecasts can be unreliable to plan for the medium- and long-term, scenario building has recently been increasingly used. However, scenario building methods often fail to take disruptions and wild cards into account, i.e., low probability but high impact events. When unaccounted for, wild card events, like the COVID-19 pandemic, lower the efficacy of scenario building in policy making, as these events may completely disturb the developed scenarios of the future.

**Methods:**

In this paper, we develop and apply a creative and participatory methodology to develop visions and disrupted scenarios for rural mobility. Our research was carried out in the Belgian village of Oetingen, where inhabitants developed more resilient views of the future by creating disrupted mobility scenarios and a preferred mobility vision for their village for the year 2050 in a participatory scenario building exercise. Wild cards related to mobility were collected from mobility experts and inhabitants in three workshops. Inhabitants were engaged to define their mobility vision on a postcard that was distributed to all houses in the village as well as on a project website. Respondents were invited for a follow-up interview in which their preferred mobility vision was subjected to the wild cards, and participants described how these wild cards would change their preferred vision. As children tend to have more creative ideas, they were engaged via workshops at school.

**Results:**

This process resulted in mobility scenarios for the village for the year 2050 based on the different wild cards, as well as an overall desired vision. We found that the use of wild cards did not significantly change the scenarios when compared to the vision, although it did make the interviewees step outside of their comfort zones. We also found that the citizens did not have more original and less path-dependent ideas in developing wild cards when compared to experts. Lastly, we found that children have many outside-of-the-box suggestions when it comes to the future. Although some of their ideas can be judged as impractical by today’s standards, many ideas had an indirect implication for mobility in the village and gave insights into children’s priorities, as potential future residents of the village.

## Introduction

Despite the fact that current transport policy is often based on forecasts, forecasts are often inaccurate [1]. Forecasting models are continuously updated,however, their accuracy has not improved [2]. This is because the future is not a linear result of the past, and it will also not be a linear continuation of the present.

Modern transport forecast methods are based on how the current situation is likely to evolve [3]. Hence, they are not equipped to handle uncertainty, and sudden changes are not considered. Recently, there has been an evolution towards non-traditional future studies such as scenario planning. Scenarios show different possible future pathways, whereas a forecast aims to predict only one. Nevertheless, traditional and path-dependent forecasting methods are still dominant [4]. For example, in their analysis of 62 recent future studies papers, Miskolczi et al. [5] found that 16 were based on scenario planning, while 46 were based on forecasts.

Scenarios are “an internally consistent view of what the future might turn out to be” ([6], p. 63). Scenario planning and decision making, however, often fail to incorporate outliers and divergent thinking. As a result, linear, non-disruptive thinking often dominates [7]. The outbreak of the COVID-19 pandemic in 2020, for example, has shown “the importance of incorporating more diverse and non-linear visions into decision-making” ([7], p. 11). Such events can have a high impact, but their probability is low, and they are often not taken into account when developing mobility scenarios. Soria-Lara et al. [7] argue that scenarios need to incorporate disruptions in order to be useful for policy making, thereby reducing linear thinking. This can be done using wild cards, i.e. events that have three characteristics: their impact is high, their probability of occurring is low, and they appear suddenly [8]. Such an event is seen as a surprise and would drastically change our lives [9]. Examples of wild cards related to transport include a drastic decrease in passenger transport and flying cars [10]. Due to their low likelihood, many wild cards will never occur. Nevertheless, identifying them can make policies more resilient to disruptions, allows the identification of early warning signals, and gives time to make investments that increase resilience [9].

Wild cards are often used to test the robustness of scenarios [7]. Von der Gracht and Darkow [11], for example, provide insights in possible changes in the macro-environment by developing logistics scenarios for 2025 and use eight expert wild cards to conduct an analysis of discontinuities. Ecola et al. [12] incorporate sustained very weak growth as a wild card event to develop scenarios for the future travel demand in China by 2030. Although no wild cards were used, Tuominen et al. [13] involved young people aged 15 to 17 in their visioning process, which resulted in visions that were more original than expert visions. Even when using more open methods, such as interviews and focus groups, linear thinking dominates the visioning process [14]. It should be noted that wild cards are a relatively new topic in foresight literature, so questions remain, such as how to best generate them or studying their impacts [10].

In addition to scenarios, visions are also used in transport policy making. Contrary to scenarios, visions provide a powerful single image of a desired future [15]. A vision is made up of two key components: (1) a desired future configuration, and 92) a future environment where this configuration can be successful [16]. The difference to scenarios is therefore that a vision represents a desired future, while scenarios map out possible futures. In terms of flexibility, multiple possible scenarios keep more options open for decision makers by providing different courses of action. Combining normative (visions) and exploratory (scenario) approaches can be considered to make the process more relevant for policy making [15].

Despite the fact that participation can increase the probability of implementing research outcomes [15], scenario building and visioning exercises are often developed top-down by experts [17]. In addition, experts are used to visualize the future linearly [18]. In our research, we developed a participatory approach, and we paid special attention to the involvement of children, as they are often not included in policy making. Children up to the age of 14 make up over 25% of the global population and 17% of the population in Belgium [19]. Children move around less and use the local surroundings more often than adults. However, children are not involved in the design of the places and spaces they use most often. In addition, younger people have been known to have a better capacity of visualizing disruptive scenarios, when compared to experts and adults [20]. They will also be the next generation of adults.

Visioning and scenario building exercises tend to focus on urban areas. These exercises are, for example, a crucial part in the development of Sustainable Urban Mobility Plans (SUMPs). SUMPs have been in use since 2013, stimulating the transition towards sustainable mobility in cities [40]. Only recently have guidelines for SUMPs in smaller cities and towns been published [41]. However, these guidelines do not apply in very low-density contexts (less than 5000 people and less than 300 people/km^2^). As rural areas are often characterized by low public transport accessibility and long waiting and travel times [21], rural communities are often car dependent [22]. Visions and scenarios can offer new and radically different ways of approaching the problem of individual mobility [23]. The focus of our research therefore lies on a rural area. Throughout our analysis of the literature, we did not find any scenario building exercises focused specifically on rural mobility. Applying these methods in a car dependent rural area thus offers an interesting avenue for research.

The goal of our research is to apply creative and participatory methods to develop a vision and different disrupted scenarios for the mobility of a Belgian village in 2050. In this paper, we seek to address the following research question:

How do citizens from rural areas imagine the future of mobility in their village using visions and disrupted scenarios?

Section 2 provides the materials and methods employed in our research. Section 3 gives an overview of the results, discussed in Sect. 4. In Sect. 5, we provide some concluding remarks and indications for further research.

## Materials and methods

The goal of our research was to develop one vision and several alternative scenarios for the Belgian rural context. Our methodology contained two main phases: i) case study selection and wild cards development, and ii) participatory visioning and scenario building.

### Case study and wild card selection

**Fig. 1:**
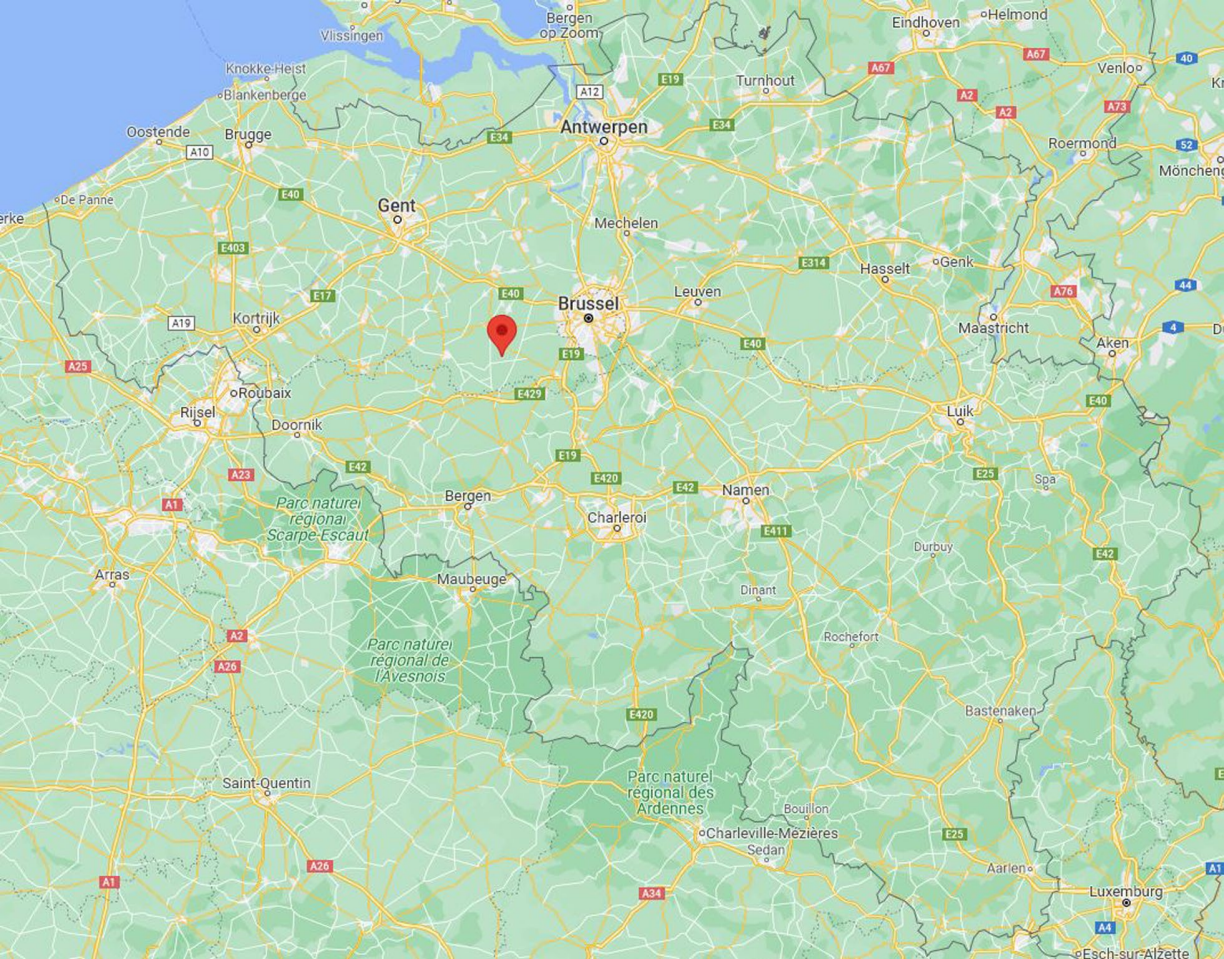
Location of Oetingen [33]

Oetingen is a Flemish village of about 2200 inhabitants located 25 km southwest of Brussels, Belgium (see Fig. 1). It is located in the Pajottenland, an agricultural part of the country [24], and is, with three other villages, part of the municipality of Gooik (9 230 inhabitants) [25]. The village was connected to Brussels via a tram, until its discontinuation in 1973. Nowadays, most residents are car dependent [26]. A bus runs every hour on weekdays, once every two hours on Saturdays, and not on Sundays. The nearest train station is 8 km away. 26% of the municipality of Gooik’s inhabitants use a bicycle for leisure, but only 10% commute by bicycle. 25% of the citizens indicate using public transport for their commute [26]. 41% of the citizens indicate walking for leisure, while 13% indicate it as a commuting mode. Public transport is used by 30% of the inhabitants to commute, but only by 15% of them as a travel mode for leisure activities. Currently, 98% of Gooik’s residents live in a household that owns a car or a van [26]. The municipality has a bike plan for 2025, aiming to become more bike-friendly. It currently does not have a mobility plan, as is often the case with smaller municipalities. The village of Oetingen was selected as it is representative for the Belgian countryside.

**Fig. 2:**
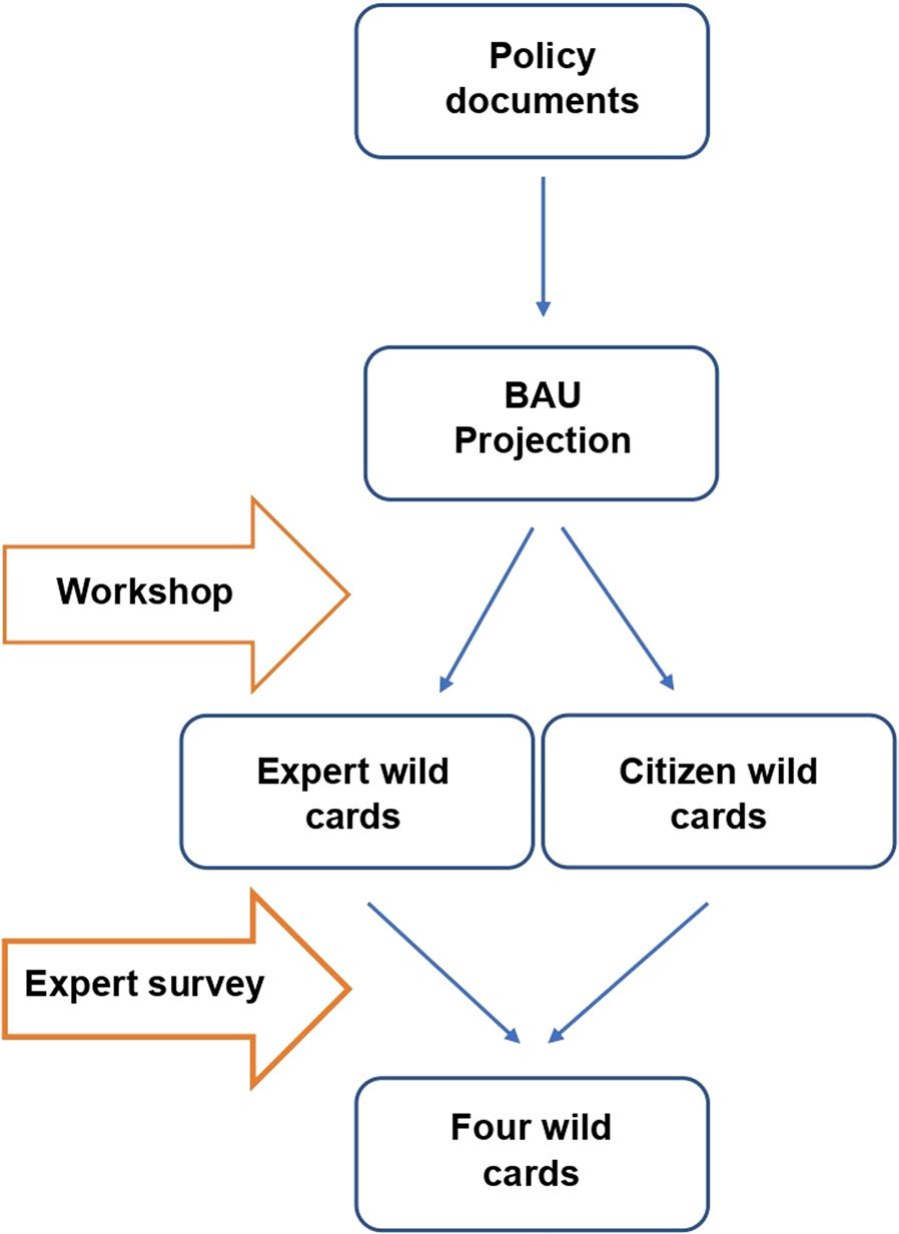
Wild card development process

After selecting the case study, we developed the wild cards to be used in the scenario building exercise. Our methodology for their development can be seen in Fig. 2.

To develop the wild cards, we held three workshops in the spring of 2021. Two were attended by academic researchers from various mobility- and transport-related fields (e.g., urban mobility, sustainable logistics, sustainable energy communities). Each workshop was attended by five experts. The third workshop was attended by the members of an Oetingen citizen association, who all are male. The lack of gender representativity can have an effect on the wild cards developed, since women’s mobility behaviours and needs are very different from men’s [27]. The goal of the workshops was to develop as many wild cards as possible. We first developed a business-as-usual (BAU) projection for 2050 for Oetingen, based on local, regional, and federal policy documents. We then asked participants to imagine wild card events that could disrupt the BAU projection. For every trend, the policy documents were consulted at the lowest political level of availability, starting from the municipal level. The BAU projection is as follows (Table 1):

**Table 1:** Oetingen 2050 BAU projection

Trend category	Trend description	Source
Social and economic	Population ages: 27% of inhabitants will be aged 67 + in 2050. (Level of availability: regional—Flanders)	[34]
	Economic development focuses on digitalisation and technological developments, and innovation will lead to autonomous, connected, multimodal, green, and shared mobility. (Level of availability: regional—Flanders)	[35]
	Proximity to businesses and services is key, also in the countryside. Villages in Gooik will therefore increase in number of inhabitants by 2050. (Level of availability: regional – Flanders; and municipal—Gooik)	[35]
Mobility and transport	Safety on roads increases: 133 deaths/year by 2050 (but not 0). For comparison: in 2020, 254 people died on Flemish roads [25]. (Level of availability: regional—Flanders)	[36]
	Cycling increases strongly, with improved infrastructure (like better connectivity between town centres). (Level of availability: municipal—Gooik)	[37]
	Decrease in level of public transport service, so private vehicles remain dominant. Public transport is only necessary for ‘core corridors’ between major economic centres; other mobility solutions will be personalised and on-demand. (Level of availability: regional—Flanders)	[36]
Climate change and decarbonization	The region will be carbon neutral by 2040, thanks to decreased energy demand and increased electrification. The EU will be climate neutral by 2050. (Level of availability: provincial—Pajottenland and supranational- EU)	[38] [39]

**Fig. 3:**
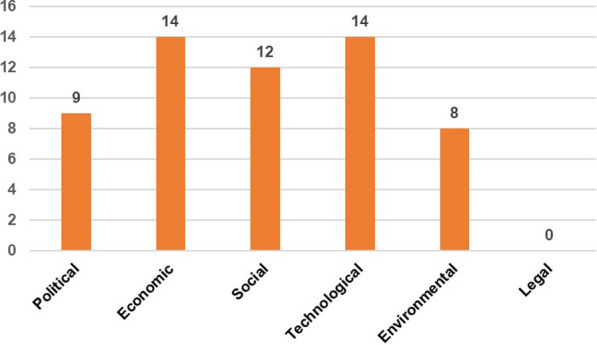
Number of wild cards per PESTEL category

The three workshops resulted in 72 wild cards. After merging duplicates that had emerged in different workshops, 57 wild cards were categorized according to the PESTEL classification (Political, Economic, Social, Technological, Environmental, Legal) to cover all relevant categories of events [28]. Figure 3 shows how many wild cards were developed in each PESTEL category. Examples of wild cards developed include ‘The end of Belgium’, ‘Increasing energy prices’, or ‘Virtual reality replaces real life’. All wild cards can be found in the annex.

**Fig. 4:**
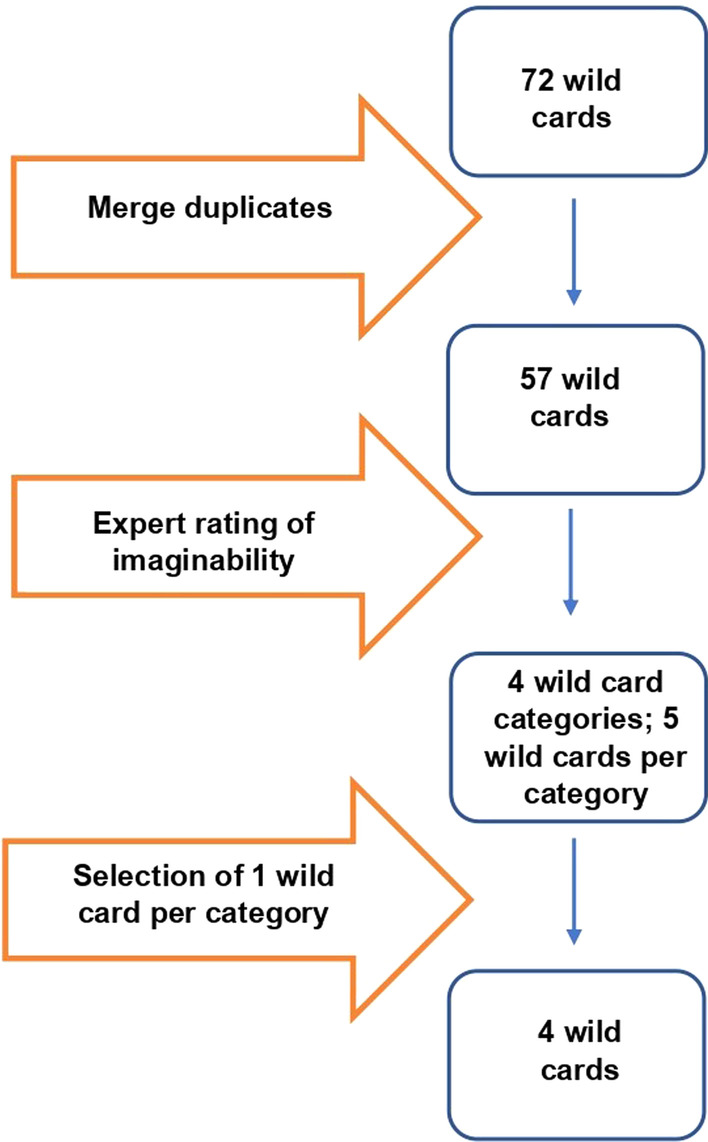
Wild card selection process

Next, we selected the wild cards to use for the scenarios. For this purpose, we circulated a survey among experts to rate the level of imaginability of the 57 remaining wild cards, i.e., their likelihood of occurrence. The scale we used is the following: very unimaginable, unimaginable, neither imaginable nor unimaginable, imaginable, and very imaginable. Unimaginable wild cards are events that constitute highly improbable surprises in the short and long term, whereas imaginable wild cards are events that constitute possible surprises [7]. In total, 24 experts from various fields filled in the survey (fields of expertise included electric mobility, logistics, forecasting, urban mobility, automated mobility, local energy systems, and battery innovation). We then identified the five wild cards in the four extreme categories (very unimaginable, unimaginable, imaginable, very imaginable) t most voted by the experts. From these five, we selected one in each of the categories to use in the scenario building exercise. Three of the selected wild cards were suggested by experts, one was suggested by citizens. The evaluation of the level of imaginability of the wild cards was done to assess whether different levels of imaginability would result in very different scenarios. The wild card selection process is depicted in *Fig. **4*.

Four wild cards were selected:

1. Very unimaginable: *‘Private cars are banned. Radical political parties view private cars as a source of inequality and ban them. Public transport is the only transport mode available.’*
2. Unimaginable: *‘The implementation of bike lanes is delayed. A car-obsessed governmental majority delays the implementation of bike lanes.’*
3. Imaginable: *‘Energy crisis results in mobility poverty. Fossil fuels are depleted and the supply of energy from renewable sources does not meet the demand.’*
4. Very imaginable: *‘Mandatory teleworking replaces all office jobs.’*

### Participatory vision and scenario development

Next, we developed the vision and the scenarios with citizens. Input for the vision was collected through postcards, the project website, and workshops with the children. The vision was then subjected to wild cards through interviews to obtain the disrupted scenarios.

**Fig. 5:**
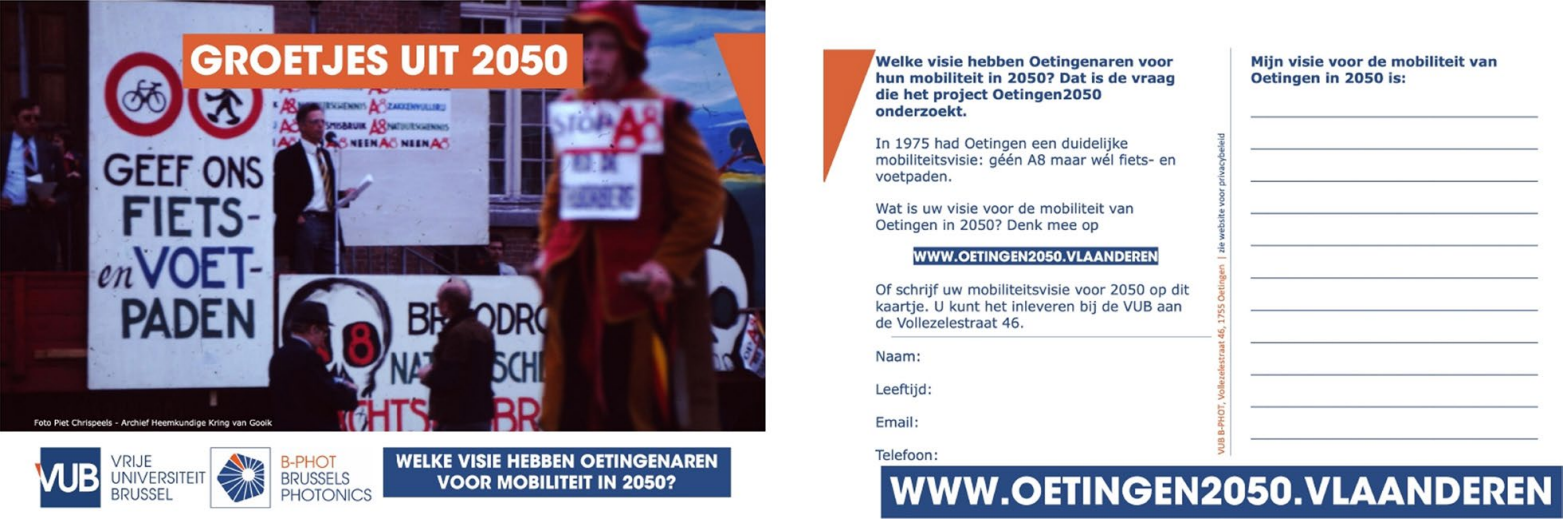
Vision postcard

To involve as many citizens as possible, participation was done both online and offline. Citizens could write their vision on the project website [29], or they could hand in a postcard that contained their vision. The postcard contained a picture from 1972 of a protest against plans for a highway. The use of a picture of a protest aimed to catch residents’ attention and increase participation. On the front, ‘Greetings from 2050’ was written, and ‘What vision do people from Oetingen have for mobility in 2050?’. The back contained more information about the project as well as space for citizens to write down their vision (see Fig. 5).

In May 2021, every household in the village received a postcard (800 postcards were distributed), which could be returned at a central location in the village. The project was also promoted via a banner on the main road, via local social media accounts, and it was covered by local media outlets. In total, 31 visions were sent in by citizens: 5 via postcard and 26 through the project website. The average age of respondents was 47.

**Fig. 6:**
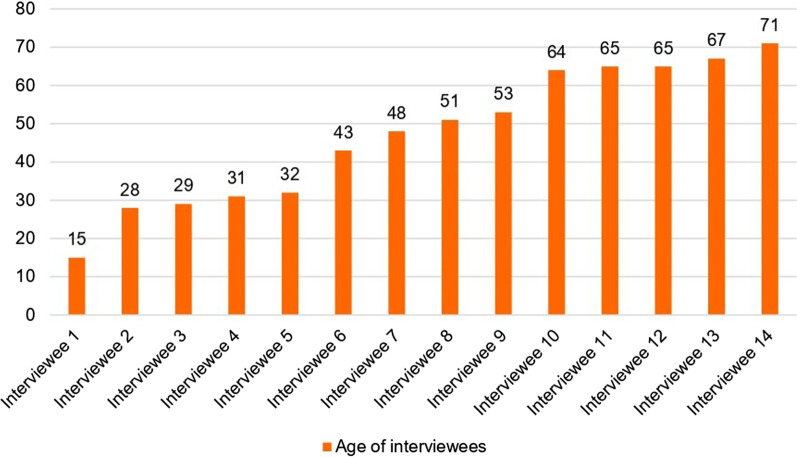
Age of interviewees

All citizens who submitted a vision, through the postcard or online, were invited for a follow-up interview to detail it further. Of the 31 people that submitted a vision, 12 responded positively to the request for a follow-up interview. 14 people were interviewed, as two of the interviewees were a couple. Ten men and four women were interviewed, with an average age of 56. The youngest interviewee was 15 years old, the oldest was 68. The distribution of the age of interviewees can be seen in Fig. 6. Semi-structured interviews were employed because there were pre-defined themes that we wanted to enquire after, but we also wanted the flexibility to explore new themes that came up during the interviews [30]. Important to note is that, overall, the participants in the research are not representative due to imbalances in age, gender, but also because of the small sample size.

#### Interviews with adults

The goal of the interviews was to acquire further details about the visions and subject the visions to the selected wild cards. Each interviewee’s vision was subjected to two of the four wild cards.

The interviews were structured into three parts:

i) **Detailing of mobility vision**
  Interviewees shared their vision for mobility and transport in Oetingen in 2050 by describing an ideal day in Oetingen in 2050. They were asked the following open-ended questions:
  - What transport mode will you use in 2050 within Oetingen?
  - How will citizens from Oetingen travel outside the village?
  - How often do you leave Oetingen in 2050 and where do you go?
  - How do people from outside Oetingen travel to the village in 2050?
  - How will you buy goods in 2050?
  - How will goods be delivered in 2050?
ii) **Disruption 1**
  Interviewees were introduced to the concept of disruptions and a first wild card event was laid out. They were then asked the same questions as in the first part of the interview, with the wild card being a ‘what if’ condition: e.g., “If private cars are banned, what transport mode will you use in 2050 within Oetingen?”
iii) **Disruption 2**
  The second wild card event was explained to the interviewees, and they were then asked the same questions as in the first part of the interview, with the wild card event being a ‘what if’ condition.

Each semi-structured interview resulted in one individual desired vision for 2050, and two individual disrupted scenarios for 2050.

The interviews took place between May and July 2021. Five interviews were held in-person; seven were organized online through Microsoft Teams. Interviews lasted between 20 and 45 min. All interviews were recorded with permission of the interviewees and later transcribed.

#### Workshops with children

Next to adult citizens, children were also included in our visioning exercise. We held five workshops with the children of the 6 grades of the Oetingen primary school, ages 5–12. Grades 5 and 6 were combined into a single workshop. In total, 113 children participated in the activity. The workshops were structured as follows:

i) **Introduction**
  Children were asked what ‘mobility’ meant to them. They were shown pictures of streets that were taken at the same location but in a different year and asked if they could detect differences (e.g., the village tram disappearing or the pedestrianisation of the Brussels city centre).
ii) **Current mobility**
  Children were asked what modes of transport they use and where they go to when using these modes.
iii) **Desired mobility** Children were asked what modes of transport they would like to use.
iv) **Inventing the transport mode of the future**
  Children were shown pictures of futuristic modes of transport such as a flying car and a Hyperloop. Then, we asked what transport mode they would like to use if they could invent one.
v) **Vision for Oetingen in 2050** Children were asked to calculate how old they will be in 2050. Then, they were asked what they would like the village to look like in 2050. The children were then divided into groups of 4 or 5 and given a map of the village on which they could write and draw their vision for the village. Lastly, each group presented their vision of the village to the class.

Due to privacy concerns and practical limitations, no recordings were made of the workshops with children. One researcher took notes while another conducted the workshops. After each workshop, the notes were completed with the drawings made during the workshop. The workshops with children were limited to the visioning exercise, as including wild cards was not possible due to time constraints.

#### Data processing

The visions, interviews, and notes on the workshops with the children were coded and analysed trough content analysis in the qualitative data analysis software Nvivo. Content analysis is used to analyse written, verbal and visual communications [31]. The results of the interviews and of the children’s workshops were coded separately. For the interviews, codes were divided into four main categories of analysis (Table 2). The ‘Vision’ code was subdivided into the following categories: i accommodation; ii eating and restaurants; iii learning; iv leisure; v mobility; vi nature; vii shopping; viii sports; ix urbanism, and x other. These same subcategories were used to code the children’s visions.

**Table 2:** Main coding categories

Code name	Description
Vision	The category ‘Vision’ contained all codes pertaining to the initial 2050 vision of all respondents (website, post cards, workshops, and interviews)
Wild card 1: Private cars are banned	This category contained all codes pertaining to the disruptions caused by wild card 1
Wild card 2: The construction of f bike lanes is delayed	This category contained all codes pertaining to the disruptions caused by wild card 2
Wild card 3: Energy crisis results in mobility poverty	This category contained all codes pertaining to the disruptions caused by wild card 3
Wild card 4: Teleworking replaces office jobs	This category contained all codes pertaining to the disruptions caused by wild card 4

Statements containing similar messages were grouped under the same code. For the vision, codes were placed under one of the ten overarching categories that had been deductively identified while pre-reading the materials. Two researchers coded half the material separately, and then exchanged materials for a new round of coding. 103 codes were developed in the separate coding rounds for the visions of the adult citizens. After these separate rounds, all coding was examined in a consensus-making round between the researchers, and two new codes were added, resulting in 105 codes in the ten overarching categories. A similar procedure was followed for the children’s visions. Here, 170 initial codes in the ten categories were brought down to 160 after analysis.

For the scenarios, 67 codes were attributed in the first round of coding. During the second round, overlapping codes were identified and merged, resulting in 57 codes remaining.

It is important to note that the vision for Oetingen in 2050 that we developed is based on all collected material, while the disrupted scenarios were constructed using only the semi-structured interviews. The vision and scenarios were constructed by identifying relevant statements, coding them, and then using these coded elements to create a narrative.

## Results

**Fig. 7:**
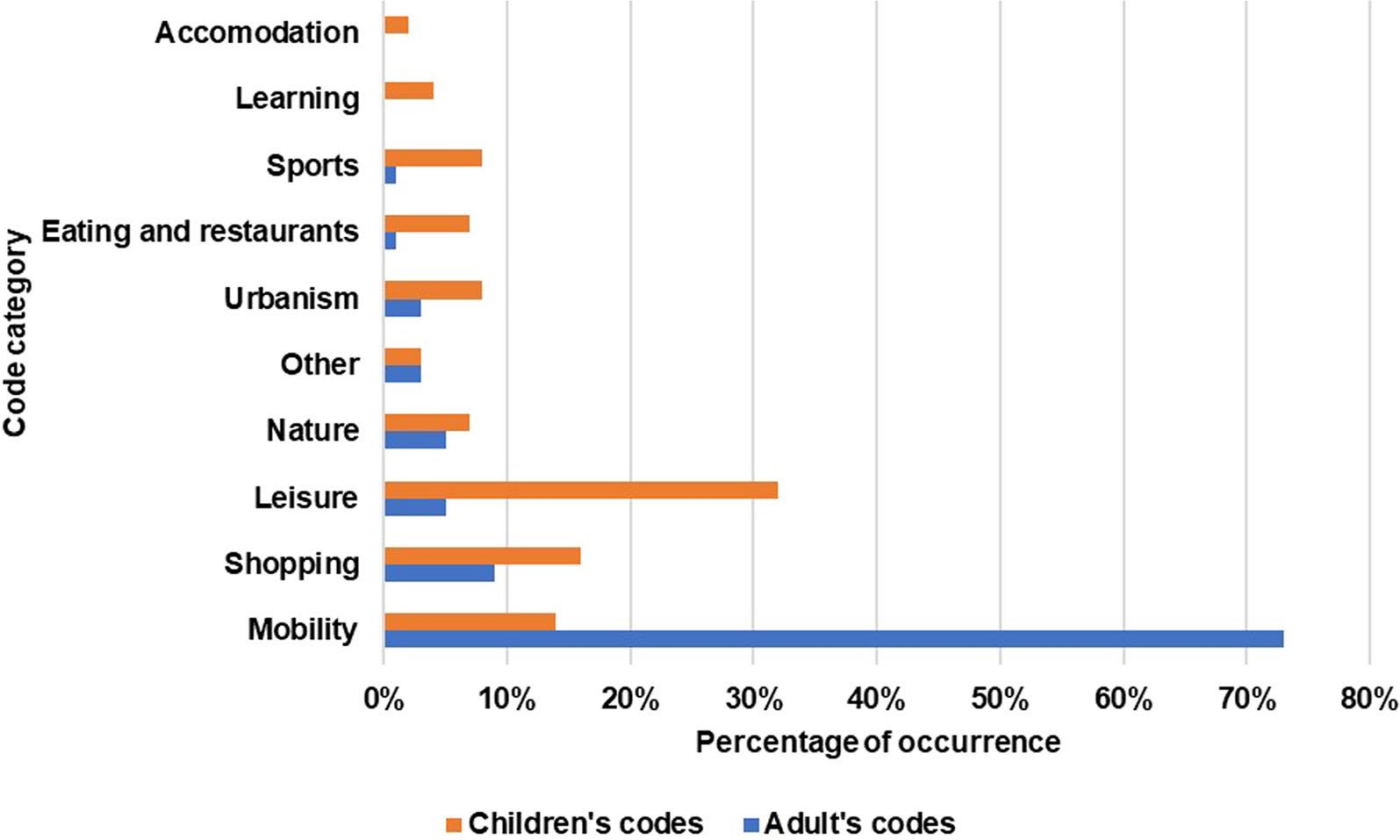
Comparison of adult and children's code categories

For the development of the vision, 31 citizens shared their input, combined with the workshops held with 113 children of the village primary school. For the development of the scenarios based on the wild cards, 12 interviews were held. In each interview, the developed vision was subjected to two wild cards, resulting in four scenarios based on the input of six interviews. In total, we developed one mobility vision for Oetingen for 2050, as well as four disrupted scenarios, each based on one of the pre-identified wild cards. The development was done by analysing the most common codes in Nvivo through content analysis. Figure 7 shows, for the visions, the difference in relative importance of all ten categories for children and adults. From the graph, we see that the most discussed theme by adults was mobility, followed by shopping. For children, most vision elements pertained to leisure, followed as well by shopping. Mobility is the third most discussed theme with the children.

Using the most recurring codes, we developed one-page narratives for the vision and each scenario. The contents of the visions and scenarios was developed based on citizens’ input and so might be unrealistic to mobility experts. However, the goal of our research was not to achieve a prediction, but to obtain an outside-the-box view based on residents’ input. Below, a short summary overview of the vision and the scenarios is provided.

### Desired collective vision for 2050: a green, tranquil yet vibrant village

In 2050, Oetingen remains a rural, green, and tranquil village. The village’s green areas have grown larger, with the addition of forests around the village centre. Citizens now move around the village mainly by (electric) bicycle, and it is a common sight to see children accompany their parents by bicycle. This can be done safely, with the car-free zones that have been established. Cycling and walking infrastructure have strongly improved as well. Walking has become an important mode of transport within the village.

Access to the centre of the village is now restricted for cars as well as heavy trucks. For trips outside the village, citizens mainly use the bus. This is possible because connectivity has improved much since 2021, with better connections to neighboring towns and cities. Public transport-based multimodality has also improved, making it easier to combine multiple buses or bicycle and bus. Many citizens still use their own private car for errands outside the village. The cars are now CO_2_-neutral, and about half of them can fly. For all electric mobility, electricity is produced by the local power station in Oetingen.

The number of trips that residents make has increased. However, the village has more shops and services, decreasing the distance of the trips. Citizens can buy local products offline without needing to leave the village. E-commerce will have gained in importance by 2050, but deliveries are more structured, with centralized pick-up points in the village. Delivery to those pick-up points is still done with trucks, but these are emission-free.

There will be more social activities in the village, further decreasing the need to leave Oetingen for such events. Children now easily spend their free time at one of the many sports fields or skate parks, or at the small local theme park. After a day spent playing or learning at one of the schools, families can go out for dinner to one of the few restaurants that have sprouted in the village.

The following paragraphs describe the four scenarios disrupted by wild cards, followed by a summary overview of how the scenarios differ from the desired vision.

### Scenario 1: private cars are banned

In 2050, there are no more private cars in Oetingen, and citizens can be seen cycling all over the town to get to their destinations. For longer trips, citizens use public transport, as connections to other towns and cities have much improved. However, as it can still be time-consuming to leave the village, individual trip frequency has strongly decreased, and people only move when it is absolutely necessary. Citizens have started using autonomous shared vehicles that operate within Oetingen and towards neighbouring towns. As planning shopping trips can be cumbersome, citizens now rely on e-commerce and home deliveries for almost all goods.

### Scenario 2: the implementation of bike lanes is delayed by a car-obsessed governmental majority

The government in 2050 is extremely supportive of private car ownership, and strongly hinders the implementation of bike lanes. As a result, Oetingen looks very similar to what it did back in 2021. People move around as much as they used to using their private cars. Bicycles are rarely used since the absence of dedicated cycling infrastructure cannot guarantee cyclists’ safety. As the traffic situation has worsened over the years, there are some attempts to develop shared mobility modes, and carpooling especially is a system that is favoured by citizens. The car is the main mode of transport used outside the village, but the cars in 2050 are all electric. As Oetingen is close to Brussels, traffic in the village has worsened due to daily commutes to and from the capital. To escape the agitation caused by the increase in traffic, some citizens have moved to more rural areas. The village is therefore less densely populated than it was in 2021.

### Scenario 3: energy crisis results in mobility poverty

An unreliable energy grid in 2050 has halted the introduction of electric vehicles. Due to their emissions of greenhouse gases, non-renewable energy sources are also not an option. Walking and cycling is now the only way for citizens to move around. There has been an increased focus towards local production and self-sufficiency, decreasing the need for citizens to leave the village. This has decreased the overall need for movement and transportation, decreasing the number of trips made by citizens, and reducing the number of visitors coming from outside Oetingen.

### Scenario 4: mandatory teleworking replaces all office jobs

As nearly all citizens in Oetingen work from home in 2050, e-commerce and home deliveries are the norm. Car ownership has drastically reduced and there are fewer cars in the streets. It is very common to see citizens cycle or walk around, and this at all hours of the day, since trips are more evenly distributed throughout the day. Mandatory teleworking has increased the feeling of community in the village in comparison with 2021, as the number of local shops and social activities have increased.

### Comparison of the vision and the scenarios

One of our goals was to see how the introduction of wild cards influences the desired 2050 vision. Table 3 provides an overview of how the desired vision and the four disrupted scenarios differ in key areas.

**Table 3:** Comparison of visions and scenarios per key element

Key elements	Vision	Scenario 1 No more private cars	Scenario 2 Delayed implementation of bike lanes	Scenario 3 Unreliable energy grid	Scenario 4 Mandatory teleworking
Mode of transport	Mainly bicycles for internal trips Mainly private (flying, emission free) cars and buses for external trips	Mainly bicycles for internal trips Public transport for external trips Autonomous shared cars for external trips	Cars are the main mode of transport There are no cyclists or pedestrians No use of public transport More shared mobility (carpooling)	No electric cars Fewer cars Bicycles are used for everything	Fewer cars Mainly bicycles or walking for internal trips
Trip frequency	Increase in no. of trips	Decrease in no. of trips	Increase in no. of trips	Decrease in no. of trips	No decrease in no. of trips, but shift in type of trips Trips more evenly distributed during the day
Shopping habits	More local businesses More e-commerce	More e-commerce	N/A	Local food production; more self-sufficiency	More local shops More e-commerce
Deliveries	Centralized pick-up points	Home deliveries of goods	N/A	No deliveries because no e-commerce	Home deliveries of goods
Village surroundings	Car-free zones and restricted heavy truck access More playground More social activities	N/A	Increased commuter traffic around the village Fewer citizens	Fewer visitors coming from outside	Increased social activities; more of a ‘village’ feeling

## Discussion

### Participation

Traditional scenario planning often remains expert-led [17]. In our research, the vision and scenarios for a Belgian village were developed based on inputs from mobility experts and citizens. The rather low number of inhabitants that submitted their vision for the village (31, or 1,4% of the population) as well as the number of interviews (12) confirms that involving the public is a challenging task, especially when there is no guarantee that decision-makers will use the outcome.

The vision and scenarios show how (a sample of) inhabitants want the village to look like in 2050. This is useful information for policymakers, as they can take decisions now that will make the village look like the inhabitants’ vision: a green and tranquil village with shops and services where inhabitants can walk and cycle. Although participation can help increase the probability of implementing the research outcomes [15], it is too early to tell whether this will be the case in Oetingen.

### Path-dependent thinking by adults

Path-dependent and linear thinking dominated the visions that were submitted via the postcards, project website, and interviews. Although 2050 is just under 30 years from 2021, all visions were slightly altered descriptions of the present. None of the suggested ideas radically challenged the status quo. While the most often used forecasting methods are path-dependent [4], our findings do not immediately confirm that more creative and participatory methods significantly reduce path-dependent thinking of adults. The wild cards did cause the interviewees to step outside their comfort zone, but most wondered what the added value was of subjecting their vision to wild cards. The changes to their vision, as seen in Table 3, were therefore not profound.

### Creative thinking by children

The visions of children were very creative and outside-the-box, including flying cars and flying bicycles. Young people have been known to have a better capacity of visualizing disruptive visions when compared to experts and adults [20]. Similarly, Tuominen et al. [13] found that involving young participants led to more original visions.

Although the children were instructed and encouraged to think of future mobility in the village, only 14% of the children’s ideas were directly related to mobility (compared to 73% of the adults’ ideas). From today’s perspective, some ideas could be considered unfeasible (e.g., a rollercoaster to move around the village), but many ideas did have indirect implications for mobility in the village. For example, an increase in leisure activities in the village will reduce the number of trips of residents outside the village but could also increase the number of trips of non-residents to the village.

With suitable methodologies, children can be involved in developing mobility visions. Their participation leads to a wide array of ideas which need to be interpreted in order to understand the meaning of what was said, or what was left out. For example, most of the children’s ideas were related to a lack of leisure infrastructure in the village, and they barely mentioned the use of cars in their visions.

### The role of the level of imaginability of a wild card

No clear link between the level of imaginability of a wild card and the contents of the scenarios was found. When comparing the vision with the scenarios, the two scenarios based on a wild card rated (very) unimaginable (scenarios 1 and 2) were not found to be fundamentally different from the scenarios based on a wild card rated (very) imaginable (scenarios 3 and 4). This is in contrast to findings by Soria-Lara et al. [7], where unimaginable wild cards lead to more disruptive thinking. Only the unimaginable wild card in which the government promotes private car ownership resulted in a scenario that was different from the vision (but similar to the current situation). The other three scenarios – no more private cars (very unimaginable),unreliable energy grid (imaginable); and mandatory teleworking (very imaginable) – resulted in scenarios that were more extreme versions of the vision. For example, increased proximity to shops and services is a key aspect of the vision that is also included in these three scenarios.

### Layman vs. expert wild cards

According to Hickman and Banister [18], experts are trained to visualize futures in a linear way. Soria-Lara and Banister [20] found that lay people have a higher disruptive visioning capacity than professionals. Our findings, however, do not confirm that lay people have more outside-the-box (i.e., unimaginable) ideas than experts. When 24 mobility experts rated the level of ‘unimaginability’ of the 57 wild cards, the experts’ wild cards were found to be more ‘unimaginable’ than those suggested by citizens. Our sample of citizens was homogenous as it consisted of five men aged 55 and over with similar socio-economic backgrounds. Soria-Lara et al. [7] found that homogeneous sub-groups of a population can be better equipped to think disruptively, but in our homogenous sample, this was not the case. However, Soria-Lara et al. [7] worked with participants between the ages of 18 and 32, which could explain the different findings.

### Rural visioning

Rural communities are often car-dependent [22], and Oetingen is no exception. This may explain why private car ownership remained dominant in the adults’ visions (10% of codes). However, cars were all but absent in the children’s visions (1% of codes). As the village is currently served by a bus, this was mentioned frequently as an alternative to private cars. Some respondents suggested a new mode of shared transport such as a light rail or monorail connection. Furthermore, while experts believe autonomous vehicles will be the norm in 2050 [32], adult citizens seemed unaware of current technological developments and the potential impact autonomous vehicles could have on their lives.

## Concluding remarks

In this paper, we presented a creative and participatory methodology to develop visions and disrupted scenarios for the mobility of a rural Belgian village in 2050. The various scenarios were developed using wild cards, as it is important to incorporate non-linear thinking into decision making [7]. Additionally, wild cards are a young topic in foresight literature, so it is important to address questions such as how to develop them, or to study their impact [10].The goal of our research was to understand how residents in rural areas in Belgium imagine the future of mobility for their village using visions and disrupted scenarios. Our aim was to showcase what elements residents find important for their future mobility, without the results needing to be realistic to mobility experts.

We involved and informed citizens in as many steps as possible. However, this involvement of lay people in the development of wild cards did not increase their level of unimaginability.

Our methodology involved people of various age categories to develop a common vision. The workshops with children resulted in very creative and often impractical ideas. However, these ideas give an interesting indication of children’s requirements. As was noted by Tuominen et al. [13] and Soria-Lara and Banister [20], the involvement of younger people leads to more outside-the-box thinking.

It is important to lower the barriers for participation. We visited the village multiple times to meet citizens and to hold interviews and offered a mix of online and offline participation methods. As most citizens were employed, we were also flexible with timing, making it easier to participate. Nevertheless, the achieved response rate among adult citizens was rather low.

It is relevant to note that our research focused on a rural area, as most scenario planning and visioning research is focused on (peri-)urban areas. As rural communities can be very car-dependent, they should not be left out of foresight exercises. Strictly speaking, our methodology is one that could also be applied to urban areas. However, the time and resources to be employed would be much higher. In the case of a village, it is possible to reach out to the entire population, and to frequently meet with citizens. In an urban context, the level of outreach we aimed for would require much more effort.

From our research, we believe it is important to include rural areas in foresight exercises for the future of transport, since these areas are usually very car-dependent [22]. Additionally, we recommend incorporating wild cards more often for the development of scenarios, since this is currently a rather research topic of research within transport [10]. Since we did not find a clear link between the level of imaginability of a wild card and the contents of the scenarios, contrary to the findings of Soria-Lara et al. [7], we recommend involving younger people in such research, to stimulate non-linear thinking.

### Limitations and future research

The research presented in this paper has limitations. Although we employed a wide array of participation methods, only 31 adult visions were submitted. We held 12 follow-up interviews. The small sample size, and the fact that participants to both the scenario building and the visioning where not representative in terms of age or gender can impact the representativeness of the vision and scenarios obtained.

Because of time constraints linked to the workshops, the children only participated in the development of the vision and not in the development of the disruptive scenarios. Future research could have children redraw their vision when subjected to a wild card. It could also be interesting to involve a more diverse number of citizens in the development of the wild cards, to increase non-linear thinking and creativity. Another direction for further research is to replicate this study in other rural areas with other characteristics (e.g., different levels of car-dependency), to analyse whether that would lead to similar or different visions and scenarios. This comparison is currently not possible since, to our knowledge, our research is one of the first to use scenario building and visioning methods in a rural context.

It should be mentioned that our research also felt the impact of the COVID-19 pandemic. ‘Mandatory teleworking’ was not as ‘wild’ as the other wild cards, as the interviews were carried out when teleworking was mandatory to stop the spread of COVID-19. The resulting scenario might therefore not be as outside-the-box as it would have been without the pandemic.

## Data Availability

The datasets generated and/or analysed during the current study are available from the corresponding author on reasonable request.

## list of selected wild cards developed by experts and citizens

See Table 4.

**Table 4:** Selected wild cards developed by experts and citizens

Category	Citizen/expert	Description of wild card	Level of imaginability (1- very unimaginable, 5- very imaginable)
Political	Expert	Private vehicles are banned Radical political parties view private vehicles as a source of inequality and ban them. Public transport is the only transport mode available	2,46
		Bike lanes are delayed A car-obsessed governmental majority delays the implementation of bike lanes	2,7
		The end of Belgium Belgium dissolves and Flanders becomes independent. A wall is built between Brussels and Flanders, affecting commuting	2,29
		The EU collapses Ambitions for climate change move to the background as more local problems need solving	2,79
		Building new houses in rural areas is no longer allowed	3,38
	Citizen	Mobility between Oetingen and Brussels becomes much easier The increased attractiveness of Oetingen poses a threat to the village atmosphere	3,96
		One-way streets All of traffic in Oetingen becomes one-way for cars	3,71
		Internal combustion engines are banned	4,33
Economic	Expert	Decrease in funds for road maintenance due to inflation Massive inflation of the euro leads to insufficient funds for road maintenance	3,17
		Economic crisis The village economy collapses, leading to high unemployment, closed shops, and citizens leaving	3,67
		An economic hub develops close to the village This increases the attractiveness of the village and causes an influx of younger, non-Belgian people	3,67
		A railway line is built through the village	3,04
		Universal basic income Universal basic income makes paid labour less necessary, thereby diminishes commuting needs	3,00
		Increasing energy prices Oil becomes scarce and electricity prices are impacted by the closure of nuclear power plants	4,38
		Increased peri-urbanisation Economic activity is located outside city centres in larger industrial zones, making it necessary to leave the city to shop	3,83
		Public transport becomes less reliable and more expensive Financial difficulties at the public transport operator. The difficulties lead to a decrease in bus lines and an increase of ticket prices	3,21
		The public transport market is opened to competition	4,21
		Explosion of the gig-economy Market liberalism results in more freelancing jobs. This causes everyone to have completely different mobility patterns	4,00
		Explosion of home deliveries Deliveries lead to unsustainable transport. When demand is too high, some areas will not get home deliveries	3,67
		Retirement is impossible Nobody can retire anymore because of the economic aftermath of COVID-19. New types of jobs will be introduced for the elderly, and the relationship with work changes for everyone	2,71
		One economic crisis per decade Three more economic crises between now and 2050 (every 10 years), with their consequences for transport demand	4,04
		Bitcoin millionaires buy up houses Rise of the bitcoin economy upturns the economic system and changes poor-rich relationships. People made rich from bitcoin start buying up houses in Oetingen	2,50
Social	Expert	Civil unrest drastically reduces transport demand Civil chaos in all of Belgium makes people avoid traveling at all costs. People are afraid to use public transport or shared mobility. They are also afraid to walk or cycle in public spaces. Only when really necessary do people travel, in private vehicles	2,46
		Population decreases due to an increase in heat waves, leading to low population density in the village	3,00
		Health issues caused by teleworking increases pressure on health care Increasing health issues related to teleworking, leaving people unable to bike. There is therefore an increasing need to get to hospitals for medical check-ups	3,08
		Urban exodus Increasing age and income gap, enhancing the rural and urban divide. Cities will become less sustainable and qualitative, leading to an exodus to the countryside	3,54
		Urban living becomes very expensive Cities become the only liveable places where proximity is still possible, so they become very expensive	3,92
		Increased housing mobility The elderly move around more and adapt their houses to their needs at a moment in time. People move out faster, so existing housing empties up for new families, but there generally is also a lower demand for traditional housing	3,83
		Collapse of the pension system The collapse of the pension system leads to increased poverty among the elderly, who cannot afford to travel and to house themselves	3,83
		Radiation fears cause exodus Growing fear about radiation emitted by the VUB photonics research centre in Oetingen results in people abandoning their houses	1,67
	Citizen	No more need for drivers' licenses because of autonomous vehicles	3,46
		Teleworking replaces all office jobs	4,25
		Decreased social capital in the village and increasing individualisation from citizens Social activities in the village and social links between villagers are reduced due to less engagement from the next generations	4,04
		People live longer and more qualitative lives You're considered old after 85 and the future elderly are in better health than the 85 + today. People remain at home longer and do not go to nursing homes. This increases the need to make all public infrastructure accessible	4,29
Technological	Expert	Virtual reality replaces real life The need for transportation is reduced drastically	2,88
		Power shortages due to increased electrification The grid cannot supply the demand	3,50
		Artificial intelligence solves all mobility problems	2,25
		Basic infrastructure is disrupted due to internet shutdowns caused by hackers or natural disasters	3,63
		Global shortages of raw materials for smart and connected applications	4,08
		Smart technologies are deemed unsafe due to hacks and weaponization	3,63
		Safety issues related to data as a commodity (like cameras, etc.)	4,17
		Home deliveries via underground tubes An underground tube transport system allows for home deliveries	2,46
		Endless and cheap energy supply Energy sources like fusion energy increase energy supply, thereby allowing for travelling without bad conscience	2,75
		Energy crisis results in mobility poverty Fossils fuels are depleted and the supply of energy from renewable sources doesn't meet the demand. This leads to increased mobility poverty due to an inability to travel as well as an increased reliance on public transport	3,54
		Oetingen becomes android-free Human-like robots (i.e., androids) are everywhere, but are increasingly hated by society. Oetingen declares itself an android-free zone, thereby becoming a walled-in community	2,13
	Citizen	Autonomous vehicles are parked outside the village	3,63
		Home deliveries are only done via drones	3,21
		All bike parkings have charging infrastructure	4,42
Environmental	Expert	The ground below Oetingen becomes unstable The instability destroys above ground infrastructure and makes it almost impossible to build above ground	2,42
		Destruction of the ozone layer People cannot walk outside without protective clothing	2,54
		Meteorites destroy Oetingen People live in shelters	1,63
		Walking and cycling impossible due to heat waves People only travel using airconditioned transport modes	3,29
		Extreme climate change Low temperatures in winter, high temperatures in summer	4,00
		Changed landscapes: energy production replaces food production The demand for energy is higher than the demand for food	3,50
	Expert & Citizen	The village is regularly under water due to flooding	3,17
		Oetingen becomes a holiday destination due to warmer weather	2,33

## list of children’s ideas

See Table 5.

**Table 5:** Overview of the children's ideas

Overarching category	Specific code
Accommodation	Camping Hotel
Eating and restaurants	Bakery Barbecue pit Donut shop Fries’ place/snack Ice cream parlour No butcher anymore Restaurants
Learning	No more school, they can learn from the couch Preschool in the village School in the village Secondary school in the village
Leisure	Animal centre/Zoo Arts and crafts place Bouncy castle Bowling Cinema Fair Farm Gaming room Horse riding club Internet and computer café Library Mini golf Museum Music school Playing on the streets Indoor playground Outdoor playground Roller coaster Scouts (Outdoor) Swimming pool Theme parc Trampoline parc (Underground) Theme parc Waterslide
Mobility	Biking Bus Cars Pedestrian crossings Emission free Flying cars Hot air balloon Walking Skateboard Skates Slide to go to school Some car-free streets Speed measurements (Underground) Train Tram Underground connections Underground parkings
Nature	Forest More green Park Water
Other	Hospital Prison Smoke-free and corona-free Time machine Veterinary
Shopping	Automated shopping Bike shop Clothing store Delivery via tubes DIY shop Drone with GPS for package delivery Food store Gun shop Market No clothing stores No supermarket One shopping street Pet shop Shopping centre Sports shop Supermarket Toy store
Sports	Formula 1 circuit for bikes Gym More inexpensive sports possibilities Motor cross circuit Mountain bike circuit No football field Skatepark Sports field
Urbanism	Airport Cemetery Fort Girls-only street Glass houses More houses No apartment buildings No high-rise buildings Power/electricity station Proximity between everything Toy factory Underground houses Villa with underground bunker
